# The Roles of Histidines and Charged Residues as Potential Triggers of a Conformational Change in the Fusion Loop of Ebola Virus Glycoprotein

**DOI:** 10.1371/journal.pone.0152527

**Published:** 2016-03-29

**Authors:** Jinwoo Lee, Sonia M. Gregory, Elizabeth A. Nelson, Judith M. White, Lukas K. Tamm

**Affiliations:** 1 Center for Membrane and Cell Physiology, University of Virginia, Charlottesville, Virginia 22908, United States of America; 2 Department of Molecular Physiology and Biological Physics, University of Virginia, Charlottesville, Virginia 22908, United States of America; 3 Department Cell Biology, University of Virginia, Charlottesville, Virginia 22908, United States of America; Division of Clinical Research, UNITED STATES

## Abstract

Ebola virus (EBOV) enters cells from late endosomes/lysosomes under mildly acidic conditions. Entry by fusion with the endosomal membrane requires the fusion loop (FL, residues 507–560) of the EBOV surface glycoprotein to undergo a pH-dependent conformational change. To find the pH trigger for this reaction we mutated multiple conserved histidines and charged and uncharged hydrophilic residues in the FL and measured their activity by liposome fusion and cell entry of virus-like particles. The FL location in the membrane was assessed by NMR using soluble and lipid-bound paramagnetic relaxation agents. While we could not identify a single residue to be alone responsible for pH triggering, we propose that a distributed pH effect over multiple residues induces the conformational change that enhances membrane insertion and triggers the fusion activity of the EBOV FL.

## Introduction

Ebola virus (EBOV) is an enveloped, negative strand RNA virus that causes severe hemorrhagic fever associated with very high fatality rates [1,2]. EBOV enters and infects cells by macropinocytosis followed by fusion of its own membrane envelope with the membrane of a late endosome [3–6]. EBOV entry and fusion are mediated by the viral surface glycoprotein (GP) [3,7,8]. GP is cleaved by cathepsins L and B in a late endosomal/lysosomal compartment, which is acidic and also contains the Niemann-Pick protein C1 (NPC1) that has been shown to be an intracellular receptor for GP [9–13]. Cathepsin cleavage, low pH, and NPC1 binding are all required to produce the active form of GP that mediates membrane fusion. After cleavage, GP is proposed to undergo a dramatic pH- and possibly NPC1-triggered conformational change that results in the exposure of the hydrophobic disulfide linked fusion loop (FL) in the N-terminal region of the GP subunit 2 (GP2) [14–16]. The low pH, but not the neutral pH form of the FL can insert into lipid bilayers in vitro and presumably also into the lipid bilayer of host endosomal membranes [17]. Insertion of the FL into the target membrane is likely mediated by a pre-hairpin intermediate consisting of the N-terminal heptad repeat region (NHR), which follows the FL, and the C-terminal heptad repeat region (CHR) is thereby linking the host and viral membranes. Folding of the NHR and CHR of GP2 into a six-helix bundle is thought to bring the two membranes into close proximity in preparation for fusion [18–20].

The fusion peptides (or FLs in the case of EBOV and several retroviruses) of class 1 viral fusion glycoproteins are essential for membrane fusion of these viruses [21,22]. Small perturbations in their sequences often produce very different fusion phenotypes [23–26]. Previous studies from our lab showed that the conformation of the EBOV FL under mildly acidic conditions, as present in the late endosome, is very different from the inactive conformation at neutral pH [15]. The FL forms a hydrophobic fist at low pH that is not present at neutral pH or when residues of a critical hydrophobic triad in the center of the fist are disrupted [17]. Although this essential conformational change has been well characterized, the pH sensor that triggers the change in shape and surface hydrophobicity of the EBOV FL is not known. Therefore, we examined in the present study numerous candidate residues in the EBOV FL in a quest to find amino acids that might be critical for sensing changes in pH and, when protonated, might trigger the conformational change of the FL.

To this end, we first examined by NMR which residues in the lipid-bound pH 5.5 conformation are surface exposed and which are buried in the hydrophobic core of dodecylphosphocholine (DPC) micelles. We next performed fusion assays on an array of EBOV FL mutants where the specific mutations were chosen based on sequence conservation and surface exposure, as informed by the NMR experiments. In brief, we examined all conserved histidines and charged residues as well as some additional hydrophilic residues that might be affected by pH-dependent hydrogen bonding. We found that histidine 516 was critical to initiate lipid mixing and leakage of liposomes, but not for viral entry. Changes in glutamate 540 and glutamate 545 were also not sufficient to alter low pH fusion of liposomes or virus entry. We conclude that the trigger for membrane fusion must be distributed over several residues in GP2, and that no simple single or double amino acid changes that we examined are sufficient to abort the pH-dependent fusion and entry activity.

## Material and Methods

### Mutagenesis, protein expression and purification

Mutagenesis, expression and purification of wild-type and mutant FLs was performed as previously described with slight modifications [15]. In brief, the EBOV FL wild-type construct (residues 507–560; derived from full-length DNA that was a gift of Dr. Paul Bates, University of Pennsylvania) was cloned into the pET41 vector (using Nde1 and Xho1 restriction sites), which contains a T7 promotor and a site conferring kanamycin resistance. Mutants were created by site-directed mutagenesis on wild-type EBOV FL using PfuUltra DNA polymerase. All constructs were grown in LB media supplemented with vitamins (Sigma product M6895 used at 1x concentration). These vitamin supplements add extra nutrients and reduce the burden on cells producing protein. This speeds up cell growth, but has a minimal effect on yield. Cell cultures grown at 37°C were induced at O.D. 600 of 0.6–0.8 with a final concentration of 0.5 mM IPTG. After introducing IPTG, cells were grown at 30°C for 4 hours and harvested. Cell pellets containing EBOV FLs were lysed by sonication for 10 min on ice and purification of FLs was performed by following the previous protocol [15].

### NMR sample preparation and spectroscopy

All NMR samples were prepared in DPC solutions as described previously [15]. For paramagnetic relaxation enhancement (PRE) experiments, stock solutions of water soluble Gd(III)-diethylenetriamine pentaacetic acid (Gd-(DTPA)), and membrane-soluble 5- and 16-doxyl steric acid (5-DSA, 16-DSA) stock were prepared. The 200 mM Gd-(DTPA) stock solution also contained 330 mM EDTA and was prepared in 30 mM sodium phosphate and 100 mM NaCl at pH 5.5 [27]. 5- and 16-DSA stocks were prepared in chloroform, dried on the bottom of glass tubes under a stream of nitrogen and resuspended by addition of 150 mM DPC to give final DSA:DPC ratios of 1:100. Wild-type FL was added to give samples of 0.5 mM FL and 150 mM DPC in 30 mM sodium phosphate and 100 mM NaCl buffer at pH 5.5. All NMR experiments were performed at 30°C on a Bruker Avance III 600 MHz spectrometer equipped with a cryo-probe. ^15^N-^1^H heteronuclear single quantum correlation (HSQC) spectra were collected and Gd-(DTPA) was titrated up to 10 mM in the final samples to observe effects of PREs from this soluble paramagnetic agent. All spectra were processed using NMRPipe and Sparky to obtain peak intensities and ratios of signals with and without the respective relaxation agents [28,29].

### Fluorescence lipid mixing assay

Lipid mixing was performed using a SpectraMaxM5 plate reader as described previously [15]. Liposomes were prepared by extrusion of lipid dispersion composed of POPC:POPG (85:15) with and without 1.5 mol% Rh-DOPE and NBD-DOPE through 100 nm pore size polycarbonate filters. Labeled and unlabeled liposomes were mixed at a ratio of 9:1 in HMA buffer (10 mM Hepes, MES, sodium acetate and 100 mM NaCl, pH 7.4) and 2.5 μM FL was added (final protein to lipid ratio, 1:20). Relief of NBD Förster resonance energy transfer (FRET) was recorded at room temperature with mixing between each scan and excitation and emission wavelengths set at 460 nm and 538 nm, respectively. Percent lipid mixing was determined as a fraction of the maximal FRET relief observed after addition of 2% Triton X-100. Data were normalized to the extent of lipid mixing observed with wild-type FL.

### Content release assay

Content release experiments were performed using a SpectraMaxM5 plate reader as described previously with slight modifications [25,30]. Liposomes containing 12.5 mM 8-aminonapthalene-1,3,6 trisulfonic acid (ANTS) and 45 mM p-xylene-bis-pyridinium bromide (DPX) were prepared by extrusion method as described in lipid mixing section using HMA buffer without salt to match inside and outside osmolarity. The dye-loaded liposomes were separated from non-encapsulated dyes by size exclusion chromatography and lipid concentrations were determined by the modified phosphate assay [31] described in Kreutzberger et al. [32].

### Virus-like particles (VLP) production and entry assay

The production of VLPs and the assay to measure their entry into cells were performed as described previously [33]. Briefly, HEK293T/17 cells (ATCC CRL-11268; obtained 8/7/13 from University of Virginia Cell Culture Core Facility) were co-transfected with plasmids encoding for VP40, β-lactamase-VP40, mCherry-VP40, and wild-type, H516A, or E540D/E545D GP with a C-terminal V5 tag (wild-type was a gift from Dr. Paul Bates, University of Pennsylvania) using the polyethylenimine method. After 48 hours incubation at 37°C, cells were centrifuged and clear media was taken and ultracentrifuged on a 20% sucrose cushion. The VLP pellets were resuspended in HM buffer (20 mM Hepes, 20 mM MES, 130 mM NaCl, pH7.4) overnight and were subsequently repelleted. All VLPs were analyzed by Western blotting for presence of EBOV GP and VP40. To measure cell entry, VLPs were allowed to enter 293AD cells (293AD cells: Stratagene #240085; obtained 10/2/14 from Stratagene) that were treated with the fluorescent CCF2-AM β-lactamase substrate for 3 hours at 37°C in the dark. Cells were washed and incubated overnight in the dark at room temperature. Cells were fixed and analyzed by flow cytometry with a FACSCalibur flow cytometer. The degree of the shift in fluorescence from green to blue was used to measure entry [34]. All data were analyzed using FlowJo software. Cells were typically passaged for not more than 30 passages before thawing and using fresh cells.

### Circular dichroism spectroscopy

All circular dichroism (CD) experiments were performed in a 2 mm path-length cuvette on an Aviv 215 spectropolarimeter. The samples contained 21 μM EBOV FL and 1 mM POPC:POPG (85:15) sonicated small unilamellar vesicles in 1 mM Hepes, MES, sodium acetate buffer initially set at pH 7.0 and containing 10 mM NaCl. The pH was titrated step-wise to the desired pH with 50 mM HCl. Samples containing 5 μM EBOV FL and 5 mM DPC in 1 mM Hepes, MES, sodium acetate buffer were prepared by dialyzing against pH 5.5.

## Results

**Fig 1:**
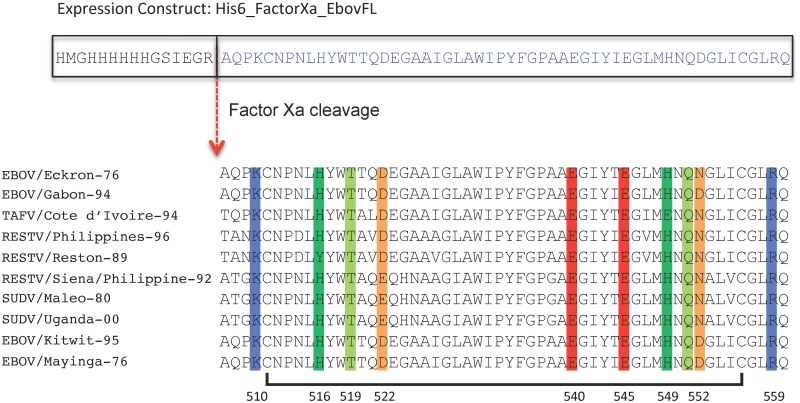
Sequence alignment of fusion loop sequences of various strains of Ebola virus. Several conserved hydrophilic residues that might serve as potential pH sensors are highlighted: dark green—histidine, light green—threonine or glutamine, blue—lysine or arginine, red glutamate, orange—aspartates (not highly conserved). The sequence of Zaire EBOV (strain Mayinga-76) was used in this study and its residue numbers and a disulfide link that defines the fusion loop are indicated at the bottom. Basic residues are shown in blue, acidic residues in red, histidines in green, hydrophilic residues in light green, and non-conserved acidic/hydrophilic residues in orange.

The disulfide-linked FL of EBOV GP2 contains two highly conserved histidines (green), two highly conserved glutamates (red), a highly conserved threonine and glutamine (both light green), two highly conserved positively charged residues (blue, just outside the loop), and two less well conserved aspartates (orange) (Fig 1). The three-dimensional structures of this loop in the presence of DPC micelles were previously determined by NMR at pH 7 and pH 5.5 [15]. In order to determine which residues were surface exposed, interfacial, or buried in the hydrophobic core of the lipid micelles we performed Paramagnetic Relaxation Enhancement (PRE) experiments in the presence of the soluble relaxation agent Gd-(DTPA) and the lipid bound relaxation agents 5-DSA and 16-DSA. The nitroxide in 5-DSA is placed closer to the micelle interface and therefore probes interfacial residues whereas the nitroxide in 16-DSA is buried more deeply in the hydrophobic core of the lipid micelles and therefore probes more deeply penetrating residues.

**Fig 2:**
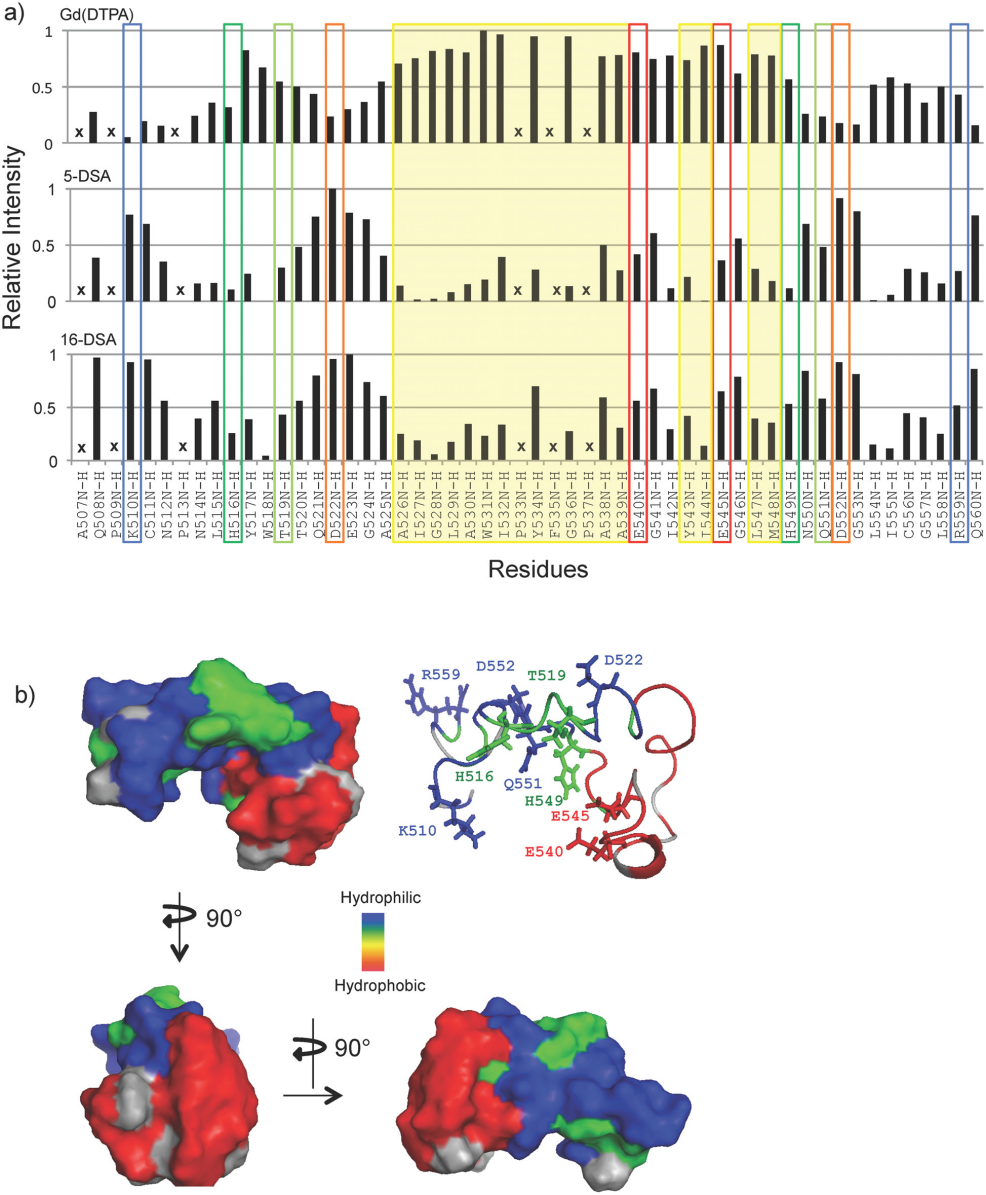
**a)** Paramagnetic relaxation enhancement NMR measurements of the EBOV FL in the presence of Gd-(DTPA), 5-DSA, and 16-DSA. The same residues are highlighted using the same color scheme as in Fig 1. In addition, residues constituting the hydrophobic patch are highlighted in yellow. Resonances of residues that are too weak to observe after quenching with the paramagnetic agents and prolines are marked with an “X”. **b)** Structure of the EBOV FL at pH 5.5 (PDB code: 2LCY) in backbone and surface representation. Residues are color-coded according to their disposition in the membrane as measured by paramagnetic relaxation NMR. Red indicates residues with deep hydrophobic and green indicates residues with interfacial lipid interactions. Blue residues are exposed to water. Grey corresponds to residues marked with an “X” in panel a.

Fig 2a shows the quenching of amide HSQC cross-peaks of the EBOV FL by all three paramagnetic relaxation agents relative to the unperturbed intensities at pH 5.5. As expected, the previously identified hydrophobic patch residues highlighted in yellow are strongly quenched by 5-DSA and 16-DSA, but not by Gd-(DTPA). As also expected, Lys510 is highly water exposed (quenched by Gd-(DTPA), but not by 5-DSA and 16-DSA) and Asp522 and Asp552 show a similar water-exposed quenching profile. 5-DSA and 16-DSA show similar, although not identical, quenching profiles. The reason for this is that the FL inserts into lipid bilayers interfacially and does not penetrate very deeply into lipid bilayers or micelles. Previous fluorescence quenching using brominated lipids and molecular dynamics simulation results in lipid bilayers showed that the FL penetrates only ~8Å in one leaflet of the bilayer [17], confirming this interpretation. In addition, the lipids in a micelle are very dynamic and the nitroxides likely visit many depths over the course of these experiments. Interestingly, Glu540 and Glu545, are much less water or lipid exposed than Asp522 and Asp552 according to the quenching data of Fig 2a, although the interfacial quencher 5-DSA is most effective for these residues. This may be due to their close spatial proximity to the hydrophobic patch in the FL. His516 and His549 are interesting because they are quenched by all quenchers and most by 5-DSA indicating their interfacial location in the micelle at pH 5.5. The hydrophilic conserved residue Thr519 shows a similar, but less well expressed interfacial quenching pattern whereas conserved Gln551 is also quenched by all quenchers, but best by Gd-(DTPA), indicating a fairly water-exposed orientation. Arg559 also appears to be more interfacial than the spatially close-by and also cationic Lys510. A summary of these data depicting the relative water and lipid exposures of most residues of the EBOV FL is shown in the surface contour plots of its micelle-bound pH 5.5 structure shown in Fig 2b, where red indicates lipid-penetrating, blue water-exposed, and green interfacial residues.

**Fig 3:**
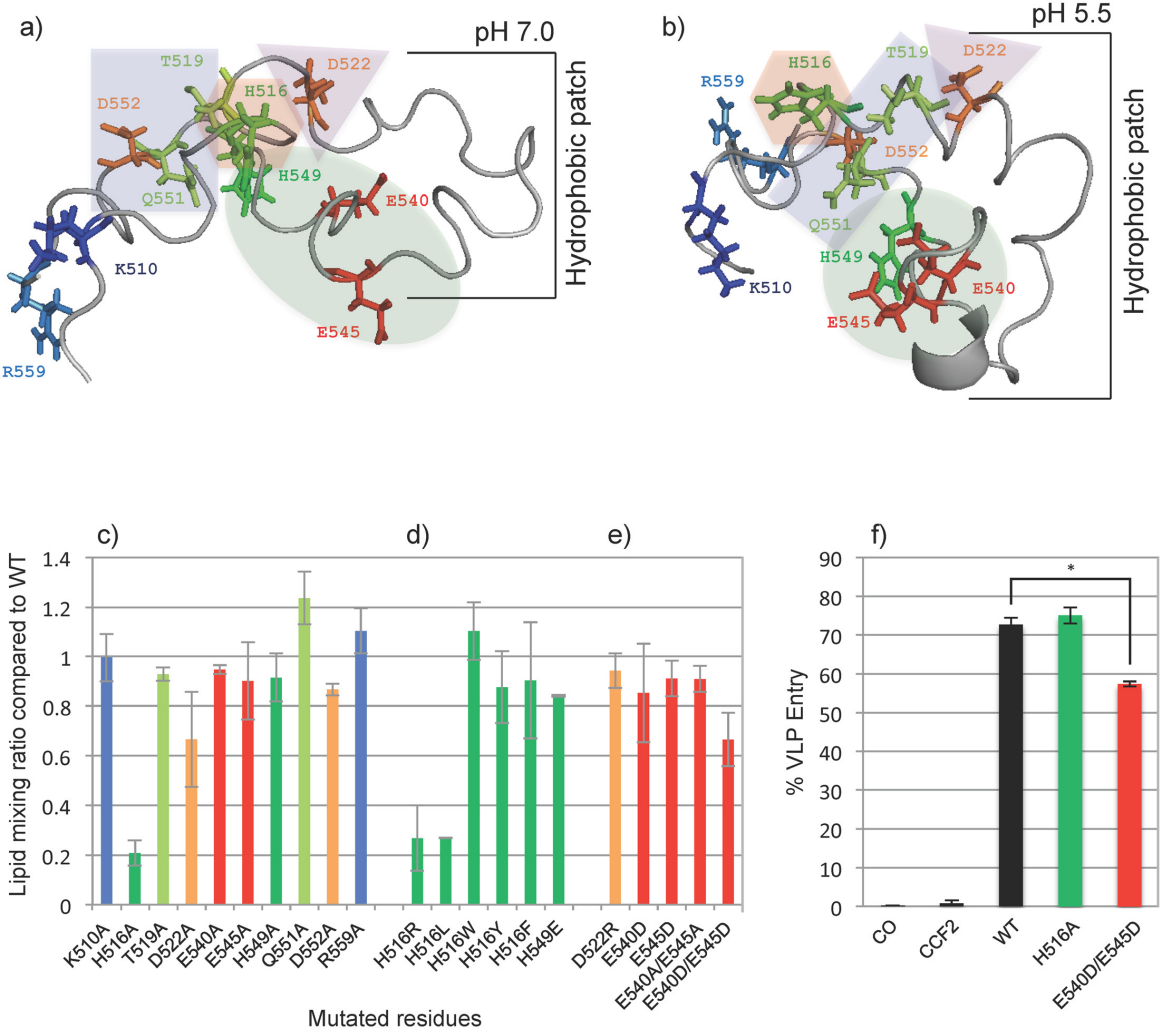
Structures of the EBOV FL at **a)** and pH 7.0 (PDB code: 2LCZ) and **b)** pH 5.5 (PDB code: 2LCY) with residues of interest shown as stick models using the same color code as in Figs 1 and 2. The residues are grouped in apparent functional clusters (see discussion): H516 or aromatic; D522 or positive charge; E540/E545/H549 group; and Thr519/Glu551/Asp552 group. **c)**–**e)** Fusion activity measured by lipid mixing of liposomes mediated by EBOV FL mutants normalized to the fusion activity of the wild-type FL. None of the mutants or wild-type FLs showed any lipid mixing activity at neutral pH. **f)** Entry of wild-type and mutant Ebola virus like particles into 293AD cells. CO—cells only, CCF2 –cells containing CCF2 but without VLPs. GP incorporation into VLPs were confirmed by Western blot and the asterisk indicates p< 0.001 in a t-test.

We hypothesized that conserved interfacial residues or residues that are partially buried in the lipid bilayer and that can be protonated at pH 5.5 would be the most likely candidates to change the structure and lipid affinity of the EBOV FL upon changing the pH from 7 to 5.5. Clearly the two histidines, but also the two highly conserved glutamates that might have shifted pKa’s because of their proximity to a hydrophobic environment would be excellent candidates to serve as pH sensors for the observed changes. Indeed, when His516 was changed to an Ala, fusion of liposomes as measured by lipid mixing was reduced to 20% of wild-type (Fig 3c). By contrast, changing His549 to an Ala had almost no effect on fusion. Glu540 and Glu545 to Ala changes also did not affect the fusion activity of the FL. The two positively charged residues that are located in close proximity at the beginning and end of the loop also had no effect on fusion when changed to alanines. Changing the conserved Thr519 or Gln551 did not affect fusion either. (Q551A exhibited a higher fusion activity than wild-type, perhaps because it made that region of the FL more hydrophobic.) The only other residue that caused some reduction of the fusion activity when changed to an alanine was Asp522 (Fig 3c). By contrast, changing Asp552 to an Ala had very little effect on fusion. The relative locations of these side chains on the structures of the FL at pH 7.0 and 5.5 are shown in Fig 3a and 3b, respectively. We also tested the ability of some of these mutant proteins to cause liposomes to leak contents. Generally, the content release assays tracked the lipid mixing assays and the effects of the same mutations on lipid mixing were greater than on content leakage from the liposomes (S1 Fig).

**Fig 4:**
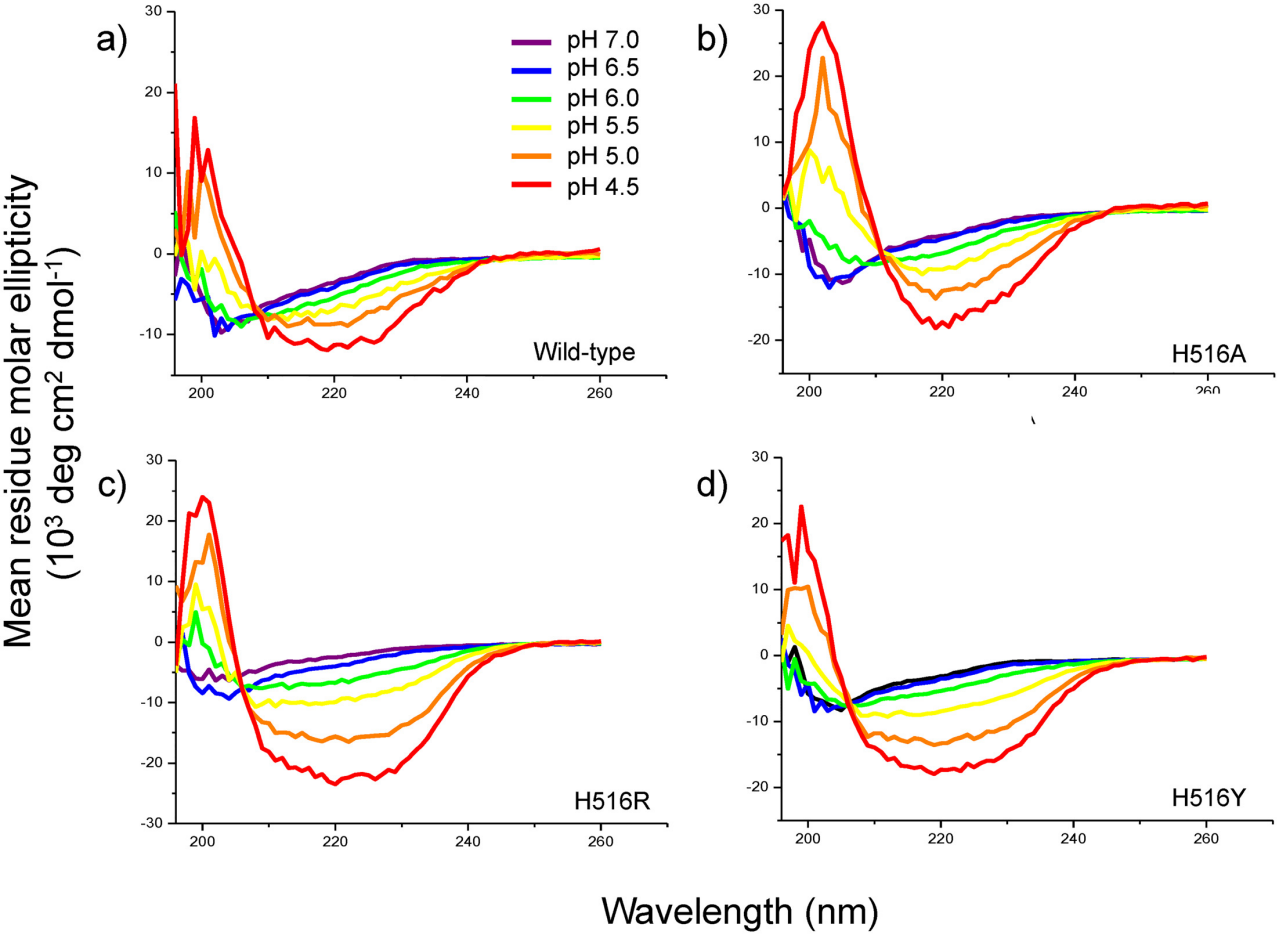
Circular dichoism spectra of the a) wild-type, b) H516A, c) H516R, and d) H516Y mutant EBOV FLs as a function of pH in POPC/POPG liposomes. The H516A and H516R mutants precipitated under acidic conditions below pH 4.5.

In order to determine if abolishment of the titratable charge on His516 or another feature in this position was responsible for the observed fusion behavior, we changed His516 into permanently charged Arg, a bulkier hydrophobic Leu, or the three aromatic residues Trp, Tyr, and Phe (Fig 3d). Fixing a positive charge on residue 516 in H516R did not recover its fusion activity. We hypothesized that His516 is protonated at low pH and mutating His516 to an arginine should keep this positive charge on residue 516 and thereby shift its pH dependency. Apparently protonation of His516 is not required to confer fusion activity to the FL. When His516 was mutated to other ring structures such as Trp, Tyr, or Phe, which all have strong tendencies to partition into membrane interfaces [35] like the native His516 does in the current structure (Fig 2), the pH-dependent fusion activity of the FL was restored. On the other hand, when His516 was replaced with the aliphatic side chain of leucine that likely pulls this residue deeper into the hydrophobic core of the bilayer, fusion was greatly diminished. Consistent with the relative insensitivity of the fusion activity to protonation of His516, the pH-dependence of the secondary structure as measured by CD spectroscopy of the EBOV FL in POPC/POPG SUVs was not dramatically changed either when this histidine was replaced by an alanine, arginine or tyrosine (Fig 4). The H516A and H516R mutations are stable at all pH values down to pH 5.0 and become somewhat cloudy after repeated CD measurements at pH 4.5 when performed at 21 μM. The H516Y mutant is stable at all pH values at a concentration of 21 μM. Thus, the CD spectra are reliable down to pH 5.0 for H516A and H516R and down to pH 4.5 for H516Y and wild-type. All wild-type NMR studies were conducted at pH 5.5 at a concentration of 100 μM or greater where they were stable. The mutant H516Y was only marginally stable and the H516A and H516R mutants were not stable under these NMR conditions, which is why we were unable to perform reliable NMR studies of these mutants. Short of such NMR measurements that could have provided residue-specific secondary structure information, we believe that the CD data support our notion that the global secondary structure of our mutants has not changed under fusion conditions, which are typically performed at pH 5.0 at a concentration of 21 μM. To further prove this point, we show in S2 Fig that the wild-type and mutant CD spectra in DPC and pH 5.5 (NMR conditions) overlay very well and the POPC vesicle data at pH 6.0 overlay reasonably well on each other. Since the histidine in position 549 is less well conserved than His516 and since in some strains a glutamate occurs in this position (Fig 1), we also changed His549 to a glutamate. Fig 3d shows that a glutamate is also tolerated in this position.

Although individual alanine mutations in E540A and E545A had no effect on fusion, the different positions of these two glutamates in the pH 7 and pH 5.5 structures (Fig 3a and 3b) suggested that protonation of one or the other residue might be sufficient to cause their reorientation and trigger pH-dependent fusion. To test for this possibility, we prepared the double mutant E540A/E545A. Even this mutant had a similar fusion activity as wild-type (Fig 3e). Since the pKa’s of aspartates are lower than those of glutamates and since this effect might be exacerbated in membrane-mimetic environments, we also prepared the single and double aspartate mutants E540D, E545D, and E540D/E545D. Fig 3e shows that single mutants showed similar fusion activities, but the double mutant exhibited a lower fusion activity at pH 5.5. This figure also shows that changing Asp522 to an Arg does not significantly change the fusion activity of this mutant, i.e., unlike the neutral alanine, a positive or negative charge in position 522 supports the fusion activity of the FL.

We also wanted to know if the same mutations built into GP1/GP2 on virus-like particles (VLPs) had an effect on their ability to enter cells. To this end, we tested the most promising pH sensor mutants H516A and E540D/E545D. Fig 3f shows that the H516A mutation did not change the entry behavior of wild-type under the tested conditions. The E540D/E545D double mutation showed a slight (~20%), but not dramatic reduction in cell entry. These results confirm that the three residues His516, Glu540 and Glu545 are not the primary pH sensors for fusion in the VLP cell entry system. Charged residues have been identified in or near the N- and C-terminal heptad repeats of the GP2 ectodomain of the related Marburg virus that stabilize the six-helix bundle structure at low vs. neutral pH [36,37]. These and other factors may be responsible for the pH sensitivity of EBOV entry. Our lipid mixing and content leakage results of Fig 3c–3e and S1 Fig, combined with our VLP entry results of Fig 3f show that fusion of liposomes mediated by the expressed FL is sensitive to additional environmental factors that play a lesser role in the VLP cell entry assay.

## Discussion

EBOV enters cells by macropinocytosis and pH-dependent fusion in late NPC1-positive endosomes. In the fusion process the GP on the viral surface as well as the membrane-interactive FL of Ebola and Marburg viruses undergo major pH-dependent conformational changes [15,38–42]. Although these changes are well documented for the GP2 subunit, the residues that trigger the conformational change upon lowering the pH are not known, neither in the complete ectodomain, nor in the FL. The goal of the current research was to determine if a clearly defined pH trigger exists in the FL and if so, which residues constitute that trigger.

Since histidines have a pKa ~6.0 when unperturbed by neighboring residues, two obvious first candidates for this trigger were the highly conserved His516 and His549 of the FL. (The former is tyrosine in the Siena/Philippine strain of Reston virus (RESTV) and the latter is a glutamate in Cote d’Ivoire strain of Tai Forest virus (TAFV). See Fig 1) Other potential residues that might trigger the pH-dependent conformational change and fusion mediated by the FL include two completely conserved glutamates and two less well-conserved aspartates. Especially glutamates may have shifted pKa’s on membrane surfaces that, when negatively charged, may attract protons increasing effective local proton concentrations near these residues and shifting their apparent pKa’s. Finally, since the completely conserved residues Thr519 and Gln551 may change their hydrogen bonding patterns in a membrane interface that accumulates protons, we also tested these residues as potential pH-triggers of FL conformation and activity changes.

To search for the pH trigger, we therefore changed all histidine, all charged, and all potentially hydrogen-bonding hydrophilic residues of the FL to alanines and in some cases to other residues, such as Arg, Leu, Trp, Tyr, Phe, Glu, Asp. The only residue that stood out in the lipid mixing fusion assay was His516, which is located at the top of the fist structure of the FL at pH 5.5 (Fig 3b). His516 showed a dramatic decrease in fusion activity when changed to a more hydrophobic residue (Ala or Leu) or a positively charged arginine.

The NMR data of the wild-type FL show that the native His in this position is located in the micelle interface and equally samples the aqueous external and hydrophobic interior face. The fusion data with mutations to the highly interfacial aromatic residues Trp, Tyr, and Phe support the notion that an interfacial location of the residue in this position is more important than its potential to be protonated at low pH or to be drawn deeper into the membrane by a hydrophobic residue in this position. However, the VLP entry assay shows that the H516A mutation, which compromised lipid mixing, was tolerated for virus entry. The highly aqueous phase exposed Asp522 was also somewhat compromised in lipid mixing when replaced by an alanine. However, reversing the charge on this residue by replacing it with an arginine was tolerated in this assay, again suggesting that positioning of the residue relative to the membrane interface may be more important for this activity than its actual charge.

The two glutamates, Glu540 and Glu545, of the FL present interesting cases. Even though they are highly conserved among different strains of EBOV, they appear to not influence low pH lipid mixing, whether they were changed individually or in tandem to uncharged alanines or negatively charged aspartates. Aspartates generally have lower pKa’s than glutamates. The double aspartate mutant showed little, but not dramatically lower fusion in the lipid mixing and in the VLP entry assay. In this case, liposome fusion mediated by the expressed FL appeared to capture the entry behavior of VLPs that display the full-length GP on their surface. It is also interesting to note that the NMR signals of these two glutamates were not strongly influenced by either the soluble or lipid-bound relaxation agents, suggesting that Glu540 and Glu545 are partially buried in a protein cavity (Fig 3b) and somewhat shielded from lipid and aqueous phase access. It is also noteworthy that Glu540 and Glu545 are far apart from each other in the pH 7 structure (Fig 3a), presumably because they are more negatively charged at pH 7. If they were partially neutralized at pH 5.5, especially in combination with a more positively charged His549 that also moves closer at the lower pH, that could explain their closer proximity and more compact fist-like structure at pH 5.5. Electrostatic repulsion of negatively charged residues has been previously suggested to be responsible for the control of pH-dependent conformational changes of several viral fusion proteins including EBOV GP [43]. Consistent with such a scenario, the shielding from NMR relaxation agents of His549 and the fusion behavior His549 mutants somewhat resemble the respective observations that we made with the two conserved glutamates.

A final group of residues concerns Thr519, Gln551, and Asp552, which are all located in a central section of the FL fist at pH 5.5 (Fig 3b). Their location in the micelle and membrane appears to be largely interfacial according to our NMR data. Neither of these residues, when changed to an alanine, changed the lipid mixing behavior of the FL, disproving the possibility that these residues, when considered individually, contributed much to the pH-dependence of the fusion behavior.

If none of the individually changed residues (or the few tested double mutants) were able to change the fusion behavior and cell entry at low pH, what other mechanism could be responsible for the observed pH-dependent fusion activity of the FL? A possible and perhaps likely scenario is that the conformational change depends on distributed, but more subtle effects of pH on multiple residues in the EBOV FL. This would be difficult to prove by simultaneous multiple mutations because cumulative mutations themselves could change the conformation of the FL in ways that may no longer represent the native conformation at neutral or mildly acidic pH. We know that the expressed FL is not very stable even in DPC micelles and, therefore, that excessive changes by mutation could obscure our ability to detect pH-triggered conformational changes. Still, the most likely residues to be subject to such distributed effects are those that we targeted in the present study.

In the case of VLP entry, which involves the entire surface GP, there are of course many more residues and interactions that potentially could contribute to the pH-sensitivity of fusion with late endosomal membranes. It is very possible that multiple still unknown pH triggers exist in full-length GP. Some may be responsible for activating the cathepsin cleavages, others may enable interactions with NPC1, and yet others may be responsible for the fusion reaction itself. Future work will need to be directed at parsing out the effects of pH on these different sub-reactions of the entry process. Differing and competing pH effects on the full-length GP may also explain the different result we obtained for the lipid mixing activity of the FL construct compared to the viral entry assay with the H516A mutant. Last but not least, the complex lipid composition in the late endosome could participate helping GP2 change its conformation at low, but not at neutral pH.

In summary, our results show that single specific residues of the EBOV FL alone are not sufficient to trigger the pH-mediated conformational change of the FL or its ability to fuse lipid bilayers. More likely, the pH-trigger for fusion is distributed over multiple residues of the FL. A most likely scenario involves an electrostatic switch including Glu540, Glu545, and His549, perhaps further aided by other hydrophilic residues and residues that must be situated in the membrane-water interface. One such residue that seems important is His516 that can be replaced by other ring structured and aromatic residues that all have a strong affinity for membrane interfaces. This general theme seems to recapitulate a similar theme that was previously observed with the much simpler fusion peptide of influenza hemagglutinin, which also bears two critical conserved glutamates, and whose interfacial structure and location in the membrane has also been shown to be important for membrane fusion and virus entry [44,45].

## Supporting Information

S1 Fig Content release from POPC/POPG liposomes mediated by mutants H516Y, H516A, H516R, and E540D/E545D relative to content release mediated by the wild-type FL. (TIFF)

S2 Fig Circular dichroism spectra of wild-type and mutant FLs in DPC and POPC/POPG liposomes. **a)** Wild-type, 516Y, H516A, H516R, and E540D/E545D in DPC micelles are compared at pH 5.5. **b)** Overlay of CD spectra of wild-type and mutants in POPC/POPG liposomes at pH 6.0 and as shown in Fig 4.
(TIFF)
