# Supplementary figures and images for: The Roles of Histidines and Charged Residues as Potential Triggers of a Conformational Change in the Fusion Loop of Ebola Virus Glycoprotein
Source: PLoS One. 2016 Mar 29;11(3):e0152527. doi: 10.1371/journal.pone.0152527 (PMC4811418; doi:10.1371/journal.pone.0152527)

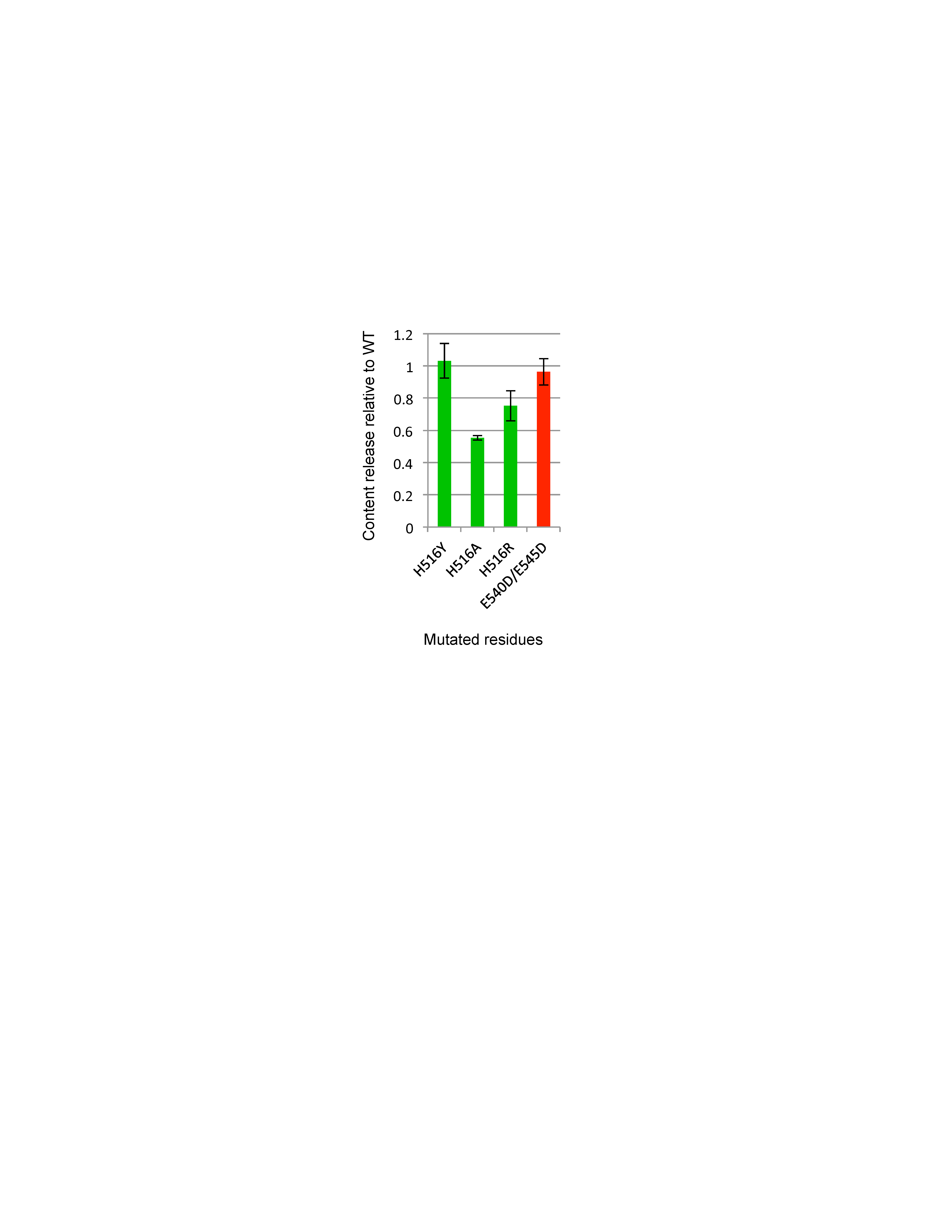

Supplement: S1 Fig — (TIFF) [file pone.0152527.s001.tiff]

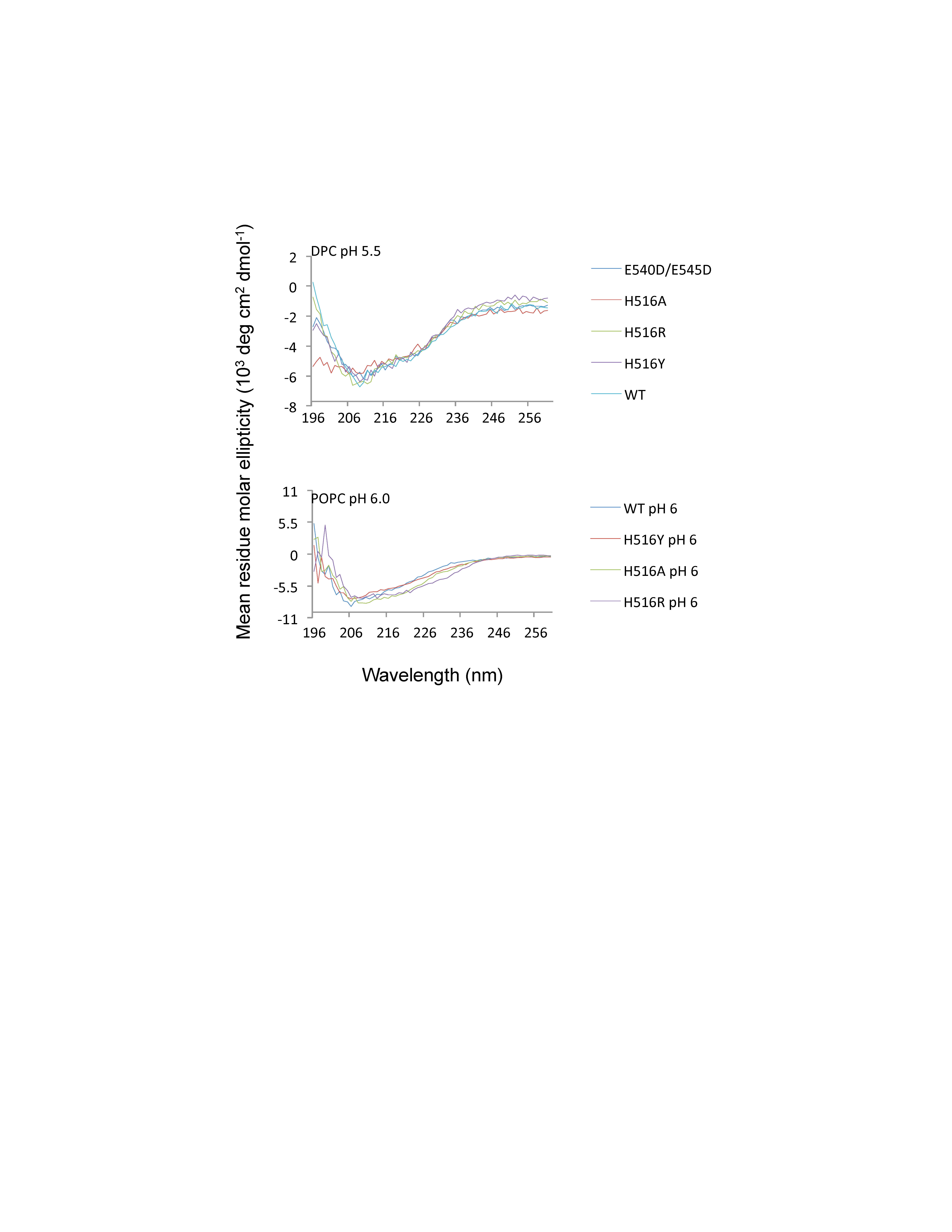

Supplement: S2 Fig — a) Wild-type, 516Y, H516A, H516R, and E540D/E545D in DPC micelles are compared at pH 5.5. b) Overlay of CD spectra of wild-type and mutants in POPC/POPG liposomes at pH 6.0 and as shown in Fig 4. (TIFF) [file pone.0152527.s002.tiff]
